# The effect of first chromosome long arm duplication on survival of endometrial carcinoma

**DOI:** 10.4274/tjod.05617

**Published:** 2014-12-15

**Authors:** Erman Sever, Emek Doğer, Yiğit Çakıroğlu, Deniz Sünnetçi, Naci Çine, Hakan Savlı, İzzet Yücesoy

**Affiliations:** 1 Kocaeli University Faculty of Medicine, Department of Obstetrics and Gynecology, Kocaeli, Turkey; 2 Kocaeli University Faculty of Medicine, Department of Medical Genetics, Kocaeli, Turkey

**Keywords:** Endometrial cancer, chromosome 1q duplication, prognosis

## Abstract

**Objective:**

The aim of this study is to investigate the effect of first chromosome long arm duplication (dup(1q)) in cases with endometrial carcinoma detected with array based comperative genomic hybridization (aCGH) on survival from the cancer.

**Materials and Methods:**

A total of 53 patients with the diagnosis of endometrial carcinom due to endometrial biopsy and who have been operated for this reason have been allocated in the study. Frozen section biopsy and staging surgery have been performed for all the cases. Samples obtained from the tumoral mass have been investigated for chromosomal aberrations with aCGH method. Kaplan-Meier and Cox-regression analysis have been performed for survival analysis.

**Results:**

Among 53 cases with endometrial carcinomas, dup(1q) was diagnosed in 14 (26.4%) of the cases. For the patient group that has been followed-up for 24 months (3-33 months), dup(1q) (p=.01), optimal cytoreduction (p<.001), lymph node positivity (p=.006), tumor stage >1 (p=.006) and presence of high risk tumor were the factors that were associated with survival. Cox-regression analysis has revealed that optimal cytoreduction was the most important prognostic factor (p=.02).

**Conclusion:**

Presence of 1q duplication can be used as a prognostic factor in the preoperative period.

## INTRODUCTION

Endometrial cancer is the most common malignant tumor of genital system in women^(1)^. Although 75% of the cases are detected while localized at early stage, recurrence of disease is observed in 15-20% of patients with endometrial cancer^(2)^. Clinical outcomes and tumor response to treatment methods which are performed on endometrial adenocarcinoma with similar stage and histological grade, may be different and emission potential of tumor can not always be estimated accurately. Therefore; new prognotic markers other than age, tumor stage, histological type and grade, lymphovascular area and lymph node involvement and tumor size, which are known prognostic factors in endometrial cancer patients, are needed^(3)^. For this reason, the effects of many genes and gene region alterations on prognosis are investigated by using advanced molecular techniques. Although still debatable, it is widely accepted that the presence of DNA aneuploidy in endometrial cancer cases estimates poor prognosis^(4)^. In addition, there are few studies investigating the relationship of gain and losses on chromosome regions with prognosis^(5,6,7,8,9)^. It was reported that aberrations of 1st chromosome, which are often seen in endometrial cancer, are associated with high grade and advanced stage endometrial cancer; however, their prognostic significance and their effects on survival have not been revealed yet^(6,7,10,11)^.

The purpose of this study is to explore the effect of the presence of chromosome 1q duplication, that is determined by array-based comparative genomic hybridization (aCGH), on prognosis in patients with endometrial cancer and its relationship with survival.

## MATERIALS AND METHODS

This study was performed with 53 cases who underwent surgical staging between April 2010-May 2011 for a diagnosis of endometrial cancer following ethics committee approval and who were willing to participate in the study with a signed consent. Both endometrioid-type and non-endometrioid histological type endometrial cancer were included in the study. Patients who have taken neo-adjuvant chemotherapy and radiotherapy were excluded from the study. All patients underwent staging surgery including total abdominal hysterectomy, bilateral salphyngoopherectomy and pelvic lymph node dissection. Cases who had more advanced stage and higher grade than stage 1A-1B and grade 1-2 tumor during frozen section examination were accepted as high risk tumor and these cases underwent paraaortic lymph node dissection. Additional surgical procedures that aim optimal cytoreduction such as omentectomy, appendectomy, peritonectomy and bowel resection were performed on cases who presented visible metastasis during surgery. Samples which were taken from endometrial tissue inside endometrial cavity under sterile conditions during surgery were frozen and stored at -80 °C for use at comparative genomic hybridization (aCGH) procedures. Presence of dup(1q) by aCGH, tumor histological subtype (endometrioid, non-endometrioid), histological grade, tumor size (<2 cm, ≥2 cm), lymphovascular area invasion, lymph node involvement and stage data based on FIGO classification were obtained from patients whose diagnosis of endometrial cancer was confirmed after pathological examination of permanent sections^(12)^. While grade 1 and 2 tumors were grouped as low grade and grade 3 tumors as high grade tumors at statistical analyses, stage 1C and more advanced stages and grade 3 tumors were categorized as high risk tumors. Stage 1 tumors were grouped as early stage and stage 2, 3 and 4 tumors were grouped as advanced stage. Optimal cytoreduction was defined as the absence of visible tumor or tumor smaller than 1 cm^3^ following surgery. Outcomes of follow up periods, survival and death due to disease were recorded following surgery.

### Detection of Deletions and Duplications

In this study, array-based comparative genomic hybridization (aCGH) method was used in order to detect deletions and duplications in tissue samples. Genomic DNA was isolated from tissue samples by using DNeasy Blood and Tissue kit (Qiagen, Hilden, Germany). DNA quality was determined by agarose gel electrophoresis and quantity was assessed by spectrophotometer (NanoDrop ND-1000; NanoDrop Technologies, Wilmington, DE). CytoChip Focus Constitutional (BlueGnome, Cambridge, UK) was used as aCGH platform. Patient DNA with a sufficient quality and quantity and reference DNA (Human Genomic DNA: Female; Promega Corporation, Madison, USA) were labelled according to CytoChip protocol. Labelled patient DNA and reference DNA were combined and they were left for hybridization at 47 °C for 20 hours with aCGH microchips according to the protocol. Microchips that were rinsed by using various dilutions of 20XSSC following hybridization period according to the protocol were scanned by Agilent Microarray scanner (Agilent Microarray Scanner; Agilent Technologies, Palo Alto, CA). Scanned images were numerically examined and all chromosomal copy number ratios were analyzed for deletions and duplications by using fixed CytoChip algorithm settings in BlueFuse Multi v2.2 (BlueGnome) software program.

### Statistical Analysis

Data of the study were analyzed by using SPSS 12 (SPSS Inc. Chicago, IL, USA). Prognostic factors affecting survival were assessed by Kaplan-Meier and Cox-regression analyses. Statistical significance was defined as p<0.05.

## RESULTS

Median age of 53 cases in the study group was 62 years (33-85). Eleven cases (20.8%) were diagnosed during premenopausal period and 42 cases (79.2%) were diagnosed during postmenopausal period. While dup (1q) was not present in 39 cases (73.6%), it was detected in 14 cases (%26.4). Significant histopathological and genetic analysis results of the cases and their surgical stages are presented in Table 1.

At the end of surgical staging, three cases (5.7%) were staged as stage 1A, 26 cases (49.1%) as stage 1B, six cases (11.3%) as stage 1C, two cases (3.8%) as stage 2A, six cases as stage 2B, three cases (5.7%) as stage 3A, four cases (7.5%) as stage 3C and three cases (5.7%) as stage 4B. Twenty-three of the cases (43.4%) had low risk tumor and 30 (56.6%) had high risk tumor. Optimal cytoreduction could be performed on 48 cases (90.6%); however, residual tumor greater than 1 cm3 was left in five cases (9.4%). Whereas adjuvant brachytherapy was performed on 30 cases (56.6%) alone or in combination with abdominal radiotherapy, nine cases (17.7%) required adjuvant chemotherapy following surgery.

Median follow up period of the cases was 24 months (3-33 months). At the end of the study, 39 cases (73.6%) were disease-free and eight cases (15.1%) survived with disease. Six cases (11.3%) died due to disease.

**Figure 1:**
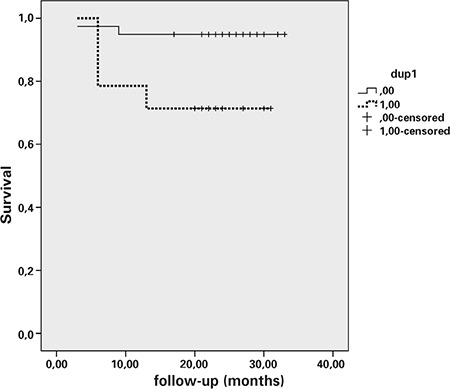
Kaplan-Meier survival anaysis of the cases with or without dup (1q) chromosomal aberration solid line-without dup (1q) and dotted line-with dup(1q)

At the end of Kaplan-Meier analysis, presence of dup(1q) (Log rank 5.51, p=.01), lymph node involvement (Log rank:11.7, p=.006), high risk tumor (Log rank 5.04, p=.02), advanced stage tumor (Log rank 7.43, p=.006) and optimal cytoreduction (Log rank 32.9, p<.0001) were the factors affecting survival (Figure 1). Cox-regression analysis revealed that the most significant indicator of survival was the availability of optimal cytoreduction among the factors tested (p=.02). When presence of dup(1q), high grade tumor and non-endometrioid histological subtype tumor, that can be determined by endometrial biopsy before surgery, were examined by Cox-regression analysis, only presence of dup(1q) was associated with survival (p=.04).

## DISCUSSION

Studies evaluating alterations in genome in endometrial cancer patients by aCGH have revealed that chromosomal aberrations often occur at chromosomes 1, 2, 3, 7, 8,10 and 12^(5,10,11,13)^. These chromosomal irregularities emerge after hyperplasies that are cancer precursors, and they are reported around 40.6% in atypical endometrial hyperplasies and at around 60-64% in endometrial cancers^(6,7)^. Most commonly seen chromosomal aberrations in hyperplasies are reductions in gene expression at 1p, 16p and 20q and elevation in gene expression at 4q^(14,15)^. Gain aberrations of long arms of chromosome 1 and 8 are frequently seen in endometrial cancer^(14,15)^. Gain aberrations of chromosome 1q were detected in 29-53% of endometrial cancer patients and gain aberrations of 8q were seen in 40% of the cases^(5,6,8,11,14,15)^. Interestingly, it was shown that gain was present at chromosome 1q among ovarian endometriosis cases presenting malignant transformation^(16)^.

In our study, we detected duplication at long arm of chromosome 1 in 14 (26.4%) of 53 cases diagnosed with endometrial cancer by aCGH technology, and we revealed the relationship of this aberration with survival. In few studies, it was reported that correlation was shown between presence of chromosomal aberrations and cellular atypia, high tumor grade, advanced tumor stage and presence of bad histological subtype^(5,6,7)^. In the studies by Sonoda et al. and Muslumanoglu et al. evaluating 14 endometrial cancer and 15 endometrial adenocarcinoma, 32 endometrial hyperplasia and 20 normal endometrial samples by CGH respectively, they have reported that chromosomal aberrations were more observed as cellular atypia, stage and grade of the tumor increased^(6,7)^. In the study by Pere et al., chromosomal aberrations were more observed in serous endometrial cancers, that are accepted as bad histological subtype, compared to endometrioid cancers^(5)^.

As in our study, regions of chromosome 1 were frequently affected in 98 patients with endometrioid cancer in the study by Levan et al. and the most frequent chromosomal alteration that they have found was gain of 1q25-->q42. In this study, gain of chromosome 1q was the most common alteration among patients who died due to cancer^(8)^. Suehiro et al. have found that copy number gains and losses during follow up were increased in cases who have died due to cancer compared to disease-free cases. In this study, it was reported that one or more copy number gains and losses of 8, 9, 11 and X chromosome regions estimated death due to disease and these aberrations showed a correlation with lymph node metastasis and cervical involvement by multivariate analyses^(9)^. In our study, when factors associated with surgery were not considered, presence of dup (1q) was significant as a prognostic factor in endometrial cancer. Interestingly, when cancer subtype, that can be detected by endometrial biopsies before the surgery, was assessed along with histological grade, it was significantly estimating death due to disease. This condition may be considered in assessing prevalence of primary disease, in revealing following recurrence of the disease and in generation of treatment plan.

Limitations of our study are few number of patients and short follow up periods. Besides, we did not evaluate chromosomal aberrations in endometrial biopsy samples by aCGH before the surgery. Therefore, we do not know whether biopsies which were performed before the surgery are sufficient to prove chromosome 1q duplication. In a study evaluating ploidy analysis in curettage or hysterectomy samples, it was reported that ploidy assessment in hysterectomy samples was superior in ploidy determination^(17)^. However, in another study, it was observed that preoperative assessment have given results as accurate as postoperative evaluation and it was useful in proving occult extrauterine emission and disease recurrence^(18)^.

In conclusion, even though chromosome studies require sophisticated techniques and they are expensive and these make it difficult to use them in clinical practice, examination of endometrial samples from endometrial cancer patients for chromosomal aberrations may be used to estimate prognosis.

## Figures and Tables

**Table 1:**
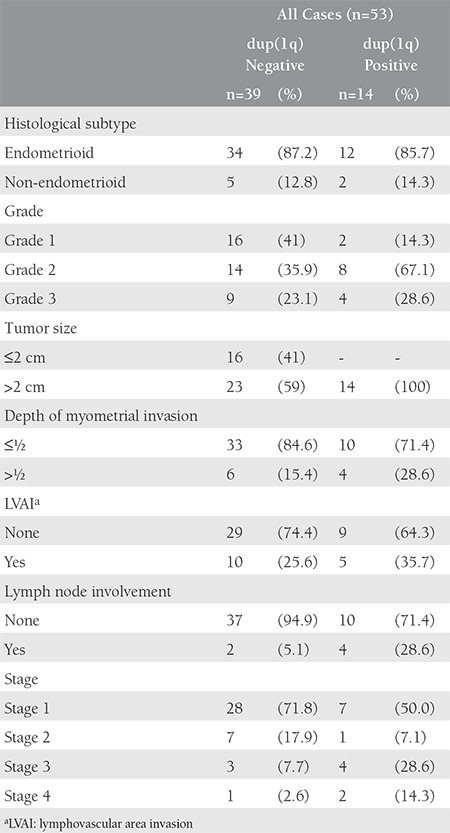
Significant histopathological and genetic analysis results of the cases and their surgical stages
